# Phytochemical Composition, Antioxidant and Antiproliferative Activities of *Citrus hystrix*, *Citrus limon*, *Citrus pyriformis,* and *Citrus microcarpa* Leaf Essential Oils against Human Cervical Cancer Cell Line

**DOI:** 10.3390/plants12010134

**Published:** 2022-12-27

**Authors:** Haneen Ibrahim Al Othman, Huda Hisham Alkatib, Atiqah Zaid, Sreenivasan Sasidharan, Siti Sarah Fazalul Rahiman, Tien Ping Lee, George Dimitrovski, Jalal T. Althakafy, Yong Foo Wong

**Affiliations:** 1 Centre for Research on Multidimensional Separation Science, School of Chemical Sciences, Universiti Sains Malaysia, George Town 11800, Penang, Malaysia; 2 Institute for Research in Molecular Medicine (INFORMM), Universiti Sains Malaysia, George Town 11800, Penang, Malaysia; 3 School of Pharmaceutical Sciences, Universiti Sains Malaysia, George Town 11800, Penang, Malaysia; 4 RCSI & UCD Malaysia Campus, 4 Jalan Sepoy Lines, George Town 10450, Penang, Malaysia; 5 Ajoya Capital Limited, World Trade Centre 1, Jl. Jenderal Sudirman Kav. 29-31, Jakarta 12920, Indonesia; 6 Department of Chemistry, Faculty of Applied Science, Umm Al-Qura University, Makkah 21955, Saudi Arabia

**Keywords:** *Citrus* spp., essential oil, GC-QMS, antioxidant, DPPH assay, antiproliferative, MTT assay

## Abstract

The essential oil derived from *Citrus* plants has long been used for medicinal purposes, due to its broad spectrum of therapeutic characteristics. To date, approximately 162 *Citrus* species have been identified, and many investigational studies have been conducted to explore the pharmacological potential of *Citrus* spp. oils. This study investigated the volatile constituents of essential oil distilled from the leaves of *C. hystrix*, *C. limon*, *C. pyriformis*, and *C. microcarpa*, using gas chromatography–quadrupole mass spectrometry. A total of 80 secondary compounds were tentatively identified, representing 84.88–97.99% of the total ion count and mainly comprising monoterpene (5.20–76.15%) and sesquiterpene (1.36–27.14%) hydrocarbons, oxygenated monoterpenes (3.91–89.52%) and sesquiterpenes (0.21–38.87%), and other minor chemical classes (0.10–0.52%). In particular, 27 compounds (1.19–39.06%) were detected across all *Citrus* species. Principal component analysis of the identified phytoconstituents and their relative quantities enabled differentiation of the *Citrus* leaf oils according to their species, with the loading variables contributing to these metabolic differences being identified. The *Citrus* leaf oils were tested for their antioxidant and antiproliferative activities using 2,2-diphenyl-1-picryl-hydrazylhydrate (DPPH) and 3-(4,5-dimethylthiazol-2-yl)-2,5-diphenyltetrazolium bromide (MTT) assays. The results indicated that *C. limon* displayed the highest DPPH radical scavenging ability (IC_50_ value of 29.14 ± 1.97 mg/mL), while *C. hystrix* exhibited the lowest activity (IC_50_ value of 279.03 ± 10.37 mg/mL). On the other hand, all the *Citrus* oils exhibit potent antiproliferative activities against the HeLa cervical cancer cell line, with IC_50_ values of 11.66 μg/mL (*C. limon*), 20.41 μg/mL (*C. microcarpa*), 25.91 μg/mL (C. *hystrix*), and 87.17 μg/mL (C. *pyriformis*).

## 1. Introduction

The genus *Citrus* belongs to the Rutaceae family, which is comprised of about 160 genera and approximately 162 species (according to the Tanaka classification system), which are distributed throughout the world [1,2]. Apart from culinary uses, *Citrus* spp. plants have been cultivated and exploited for their nutritional and therapeutic value. In particular, the aromatic oil extracted from these plants is valued due to its medicinal properties and economic significance, with potential applications in the food, perfume, cosmetic, and pharmaceutical industries [3,4]. *Citrus* spp. essential oils (EO) have reportedly exhibited various therapeutic properties and/or beneficial pharmacological effects, such as antibacterial [5,6], anticancer [7,8], anti-inflammatory [9,10], and antioxidant [11,12,13] activities. It is worth noting that these associated bioactivities and their organoleptic properties are attributed to the complex pool of phytoconstituents in *Citrus* EO, which vary according to genetic variability and diversity, origin, climate, seasonal factors, and others [14].

The phytochemistry of *Citrus* spp. EOs is comprised of monoterpenic and sesquiterpenic hydrocarbons, as well as their oxygenated derivatives, which include alcohols, aldehydes, ketones, esters, and others [15]. The characteristic EO profiles of *Citrus* spp. have been reported to identify species/cultivars, determine genetic diversity, and interpret sensory attributes [16]. Given the availability of *Citrus* plants around the world and their associated pharmacological potentials, numerous studies have targeted investigating the chemical composition of EOs derived from different *Citrus* species and/or varieties that confer distinct pharmacological properties [17]. Notably, cancer has become a focus of attention, as one of the most critical diseases and the second highest cause of death after heart disease [18]. There are many reported causes of cancer (e.g., physiological and biochemical factors), which include ultraviolet rays, smoke, poisoning by bacteria and fungi, and the presence of free radicals that cause oxidative damage to vital biomolecules [19]. Therefore, the search for cures from plant-derived products (e.g., essential oil) has become an important subject [20,21]. Considerable evidence has demonstrated that selected *Citrus* spp. EOs exhibit an antioxidant potential that can eliminate excess free radicals (e.g., hydroxyl radicals, peroxide radicals, and super oxygen radicals) in the human body [22]. Some recent studies have also highlighted the chemopreventive potential of some *Citrus* spp. EOs that displayed cytotoxicity effects against cervical (HeLa), breast (MCF-7), kidney (HEK-293), colon (HT-29), and liver (HepG2, BEL-7402) cancer cell lines [23,24,25,26].

To our knowledge, reports that have comprehensively examined the chemical constituents and pharmacological potential of EOs derived from the leaf of *Citrus* spp. plants are still lacking. Considering these points, this study aimed to (i) profile steam-distilled leaf essential oil from four commercially cultivated *Citrus* species (*C. hystrix*, *C. limon*, *C. pyriformis*, and *C. microcarpa*) in Malaysia; (ii) determine the antioxidant activities of *Citrus* spp. EOs using the 2,2-diphenyl-1-picryl-hydrazylhydrate (DPPH) radical scavenging method; and (iii) evaluate the in vitro antiproliferative activities of these oils against human adenocarcinoma cervical cancer (HeLa) cell line. The phytocomplexity and variation of the secondary compounds within different *Citrus* spp. leaf oils that may contribute to different biological effects are discussed. Discriminations based on chemometrics analyses, to objectively compare the fundamental similarities and differences among the different *Citrus* spp. are presented.

## 2. Results and Discussion

### 2.1. Phytochemical Compositions

**Figure 1:**
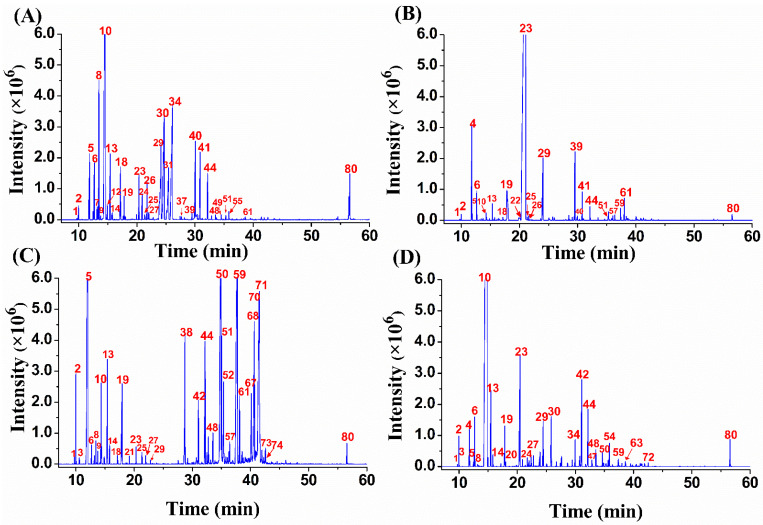
Total ion chromatograms (TICs) of four *Citrus* spp. leaf essential oils. (**A**) *C. limon*; (**B**) *C. hystrix*; (**C**) *C. microcarpa*; and (**D**) *C. pyriformis*. The numbering of the phytoconstituents is provided in Table S1 (Supplementary Information).

GC-MS analysis enabled the tentative identification of 80 secondary compounds (46 in *C. hystrix*, 52 in *C. microcarpa*, 54 in *C. pyriformis*, and 45 in *C. limon*), which represented between 84.88% and 97.99% of the total ion count within the four *Citrus* spp. leaf EOs. The chromatographic profiles of secondary compounds in the different *Citrus* spp. leaf oils are presented in Figure 1, highlighting distinctive chemical patterns. These tentatively identified compounds are distributed into 15 monoterpene hydrocarbons, 22 oxygenated monoterpenes, 16 sesquiterpene hydrocarbons, 19 oxygenated sesquiterpenes, 1 diterpenes, 1 oxygenated diterpene, and others (6). A total of 26 compounds were detected in common across all species, comprising mainly monoterpene hydrocarbons (0.01–70.40%). Table S1 (Supplementary Information) summarizes the identified secondary compounds and their relative abundance based on total ion counts. The degree of variation in the metabolic compositions (Figure 1 and Table S1) corresponding to different *Citrus* spp. EOs is noteworthy, which could be translated into differences in the expression of their pharmacological potential.

**Figure 2:**
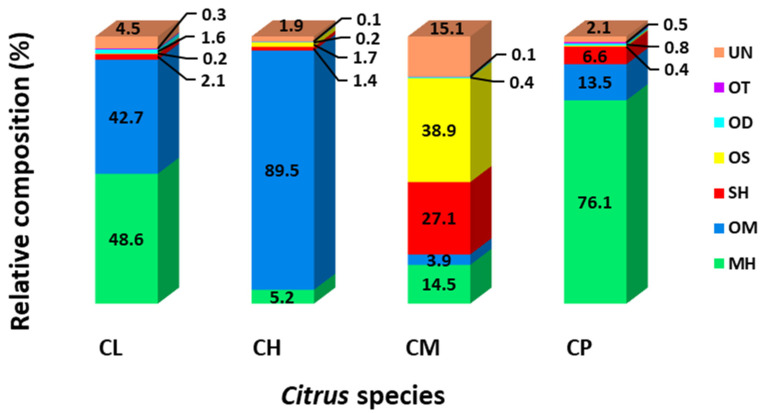
Relative phytochemical compositions (%) of the analyzed *Citrus* spp. leaf essential oils: *C. limon* (CL), *C. hystrix* (CH)*, C. microcarpa* (CM), and *C. pyriformis* (CP). The constituents included monoterpenic hydrocarbon (MH), oxygenated monoterpenes (OM), sesquiterpenic hydrocarbon (SH), oxygenated sesquiterpene (OS), oxygenated diterpenes (OD), other subgroups of metabolites (OT), and unidentified compounds (UN).

The identified major compounds (relative abundance > 1%) for different species of *Citrus* leaf oils are detailed in Table 1, while the relative composition of the classified chemical groups is illustrated in Figure 2. Notably, monoterpenic compounds predominated in *C. hystrix*, *C. limon*, and *C. pyriformis*, with total ion count abundances of 94.7%, 91.4%, and 89.7%, respectively. Interestingly, the content of monoterpenes (18.4%) in *C. microcarpa* was much lower compared to the other species (≥89.7%). Monoterpenic hydrocarbons constituted the highest proportion in *C. limon* (48.6%) and *C. pyriformis* (76.1%), as compared to *C. hystrix* (5.2%) and *C. microcarpa* (14.5%). Limonene was the major compound in *C. limon* and *C. pyriformis*, accounting for 33.6% and 70.4% of the total ion count, but only constituting a relatively small amount in *C. microcarpa* (1.7%) and *C. hystrix* (0.2%). *C. hystrix* had the highest content of oxygenated monoterpenes (89.5%), with citronellal being the principal constituent for *C. hystrix* (77.7%), while *C. microcarpa* showed the lowest amount of oxygenated monoterpenes (3.9%). Apart from *C. hystrix*, *Eucalyptus citriodora* leaf and *Litsea cubeba* fruit EOs have also been reported to contain significant amounts of citronellal, with relative composition > 50% [27,28]. This monoterpenoid aldehyde is appreciated for its effective mosquito-repelling properties [29]. Interestingly, sabinene (3.0%) and citronellol acetate (2.8%) were only present in *C. hystrix*, while β-myrcene (1.8%), 3-carene (5.4%), terpinolene (1.5%), and nerol acetate (3.6%) were only found in *C. limon*. Other identified major monoterpenes include β-pinene (0.1–7.1%), β-ocimene (0.5–2.4%), β-citronellol (0.02–5.9%), linalool (0.7–3.2%), and others.

The sesquiterpenic profiles revealed notable differences between the four *Citrus* spp. leaf oils. The contents of sesquiterpene hydrocarbons (1.4–6.6%) and oxygenated sesquiterpenes (0.2–1.7%) were relatively low in all *Citrus* spp. EOs, except *C. microcarpa* (27.1% and 38.9%, respectively). Caryophyllene (1.5%) and β-bisabolene (0.2%) were the main sesquiterpenoids in *C. limon*, while bicyclogermacrene (0.3%) and caryophyllene (0.5%) were major contributors of sesquiterpenes for *C. hystrix*. β-elemene (2.8%) and α-farnesene (0.7%) were the main compounds of these C15 fractions in *C. pyriformis*. The major sesquiterpenes identified in *C. microcarpa* were elemol (16.7%), germacrene D (13.0%), β-eudesmol (8.6%), γ-eudesmol (5.7%), α-eudesmol (3.6%), caryophyllene (3.3%), δ-elemene (3.2%), β-selinene (2.4%), bicyclogermacrene (2.0%), and nerolidol (1.5%). Interestingly, α-, β- and γ-eudesmol were not found in other *Citrus* leaf EOs, suggesting the possibility of differentiation using the presence of these secondary compounds. Phytol was identified as the only oxygenated diterpenes detected across all *Citrus* leaf oils, with relative composition of 0.2–1.6%. Overall, the phytochemical compositions of the major compounds agreed with previously reported studies [16,30]; however, variations were observed for the minor constituents across all species. This observed discrepancy may be due to the influence of several factors, such as geographical origins, climate, and others [14]. Although bioactivity is generally attributed to the major plant constituents, studies have shown that it can be enhanced by the synergistic effects of other phytoconstituents [31].

### 2.2. Discrimination via Principal Component Analysis

**Figure 3:**
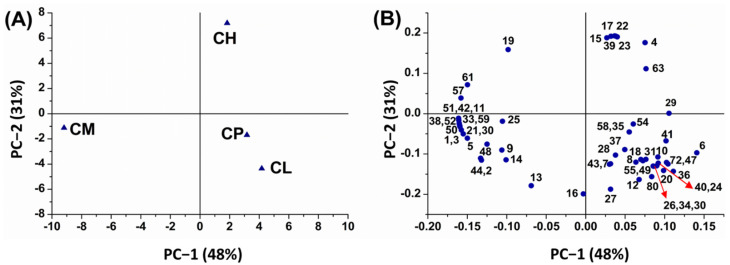
Principal component analysis (PCA) of the identified volatile compounds in leaf essential oils derived from four *Citrus* species. (**A**) Score plot, and (**B**) loading plot. *C. hystrix* (CH), *C. limon* (CL), *C. pyriformis* (CP), and *C. microcarpa* (CM). The peak numbering refers to Table S1 (Supplementary Information).

In order to examine the metabolic differences among the different species of *Citrus* leaf oils, principal component analysis (PCA) was applied to the chemical abundances of the 80 compounds identified in respective species. Figure 3A illustrates a score plot for the different *Citrus* spp. leaf oils, highlighting the potential for chemotaxonomic classification based on species. PC-1 produced the highest variation (48%) of data, followed by PC-2 (31%), which cumulatively explained 79% of the variance in the dataset. The analyzed leaf oils were segregated into four different groups, revealing discriminating secondary compounds according to the *Citrus* species, which could be explained by the loading plot (Figure 3B). It is worth noting that PC-2 separated *C. limon* and *C. pyriformis* from *C. hystrix*, and the proximity of *C. limon* and *C. pyriformis* as situated in the same quadrant (negative PC-2 axis) suggests a certain degree of similarity with respect to their secondary compounds. The main explanatory variables were trans-linalool oxide, 4-thujanol, citronellal, and citronellol acetate for *C. hystrix*; cosmene, α-thujene, camphene, β-pinene, and humulene for *C. microcarpa*; geranyl propionate, β-bisabolene, 3-carene, terpinolene, trans-β-ocimene, geranyl acetate, and phytol for *C. limon*; and trans-sesquisabinene hydrate, *p*-vinylguaiacol, and α-farnesene for *C. pyriformis.*

### 2.3. Antioxidant Activity

The antioxidant capacity of the *Citrus* leaf EOs was investigated by evaluating their DPPH free-radical scavenging ability. The citrus oils displayed a dose-dependent scavenging activity against DPPH. Based on the linear curve plotted between the scavenging activity inhibition percentage and the EO concentrations, *C. limon* exhibited the highest antioxidant activity (IC_50_ value of 29.14 ± 1.97 mg/mL), while *C. hystrix* had the lowest antioxidant activity (IC_50_ value of 279.03 ± 10.37 mg/mL). Additionally, *C. limon* and *C. pyriformis* leaf oils showed the highest DPPH radical scavenging ability, as compared to the positive control (ascorbic acid). The IC_50_ values of the antioxidant activity of *Citrus* oils and values reported from previous studies are shown in Table 2. The current results demonstrated that the antioxidant potential of *Citrus* oils might be attributed to the variations in their phytoconstituents [32]. A few studies have reported that antioxidant activity might be correlated to the level or proportion of limonene within *Citrus* EOs [11,33,34]. Overall, the current findings indicated that the antioxidant activity was higher in oils containing a higher proportion of limonene. However, the inter-relation of the limonene content and antioxidant potential of *Citrus* oil necessitates further investigation, as conflicting reports have been made [35]. Thus, it is difficult to explain how *Citrus* oil functions as an antioxidant, due to the intricacy of their multicomponent mixes and insufficient studies on their molecular mechanisms.

### 2.4. Antiproliferative Evaluation

**Figure 4:**
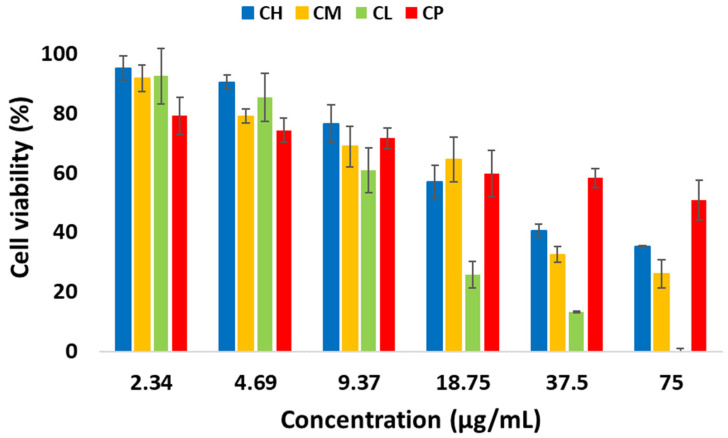
Antiproliferative activity of the four *Citrus* spp. leaf oils against the HeLa cervical cancer cell line. *C. hystrix* (CH), *C. limon* (CL), *C. pyriformis* (CP), and *C. microcarpa* (CM). Values are means of three replicates ± standard deviation. *p*-values < 0.01, as determined using one-way ANOVA.

The 3-(4,5-dimethylthiazol-2-yl)-2,5-diphenyltetrazolium bromide (MTT) assay was used to evaluate the cytotoxicity of *Citrus* spp. EOs against a human adenocarcinoma cervical cancer (HeLa) cell line, with a concentration range of 2.3 to 75.0 μg/mL. Figure 4 indicates that all *Citrus* oils decreased the viability of the Hela cells in a dose-dependent manner (*p* < 0.01). After 24 h of treatment, *C. limon* oil (IC_50_ = 11.7 μg/mL) exhibited the most significant effect, followed by *C. microcarpa* (IC_50_ = 20.4 μg/mL) and *C. hystrix* (IC_50_ = 25.9 μg/mL), while the least potent was *C. pyriformis* (IC_50_ = 87.2 μg/mL). To our knowledge, this is the first study to report a comparative investigation of the antiproliferative activities in *C. microcarpa*, *C. hystrix*, and *C. pyriformis* leaf oils. Interestingly, the results indicated no correlation was observed between the antioxidant activity (*C. hystrix* < *C. microcarpa* < *C. pyriformis* < *C. limon*) and the antiproliferative effects (*C. pyriformis* < *C. hystrix* < *C. microcarpa* < *C. limon*) against HeLa cells. The cytotoxicity of *C. limon* oil was observed to be notably higher compared to previous studies concerning different plant parts and origins. For instance, the *in vitro* cytotoxicity activity of *C. limon* peel oil from Northern Egypt against the HeLa cell line resulted in an IC_50_ value of 51.0 μg/mL, and Iranian *C. limon* peel oil displayed an IC_50_ value of 17.0 μg/mL [23,40]. On the contrary, *C. limon* leaf oil from India presented a much higher cytotoxicity (IC_50_ = 4.75 μg/mL) compared to the current study [41]. Interestingly, the antiproliferative activities of the analyzed *Citrus* leaf EOs were comparable to cytotoxic studies using *Citrus* spp. peel EOs with IC_50_ values <100 μg/mL. For instance, *C. hystrix* peel EO displayed potent cytotoxic activity against human melanoma cells (WM793, A375, and HTB140) with IC_50_ values of 59.2–88.4 μg/mL, while *C. limon* peel EO was found to be cytotoxic to HepG2 cells and HCT-116 colorectal carcinoma cells (IC_50_ value of 48.2 and 72.6 μg/mL, respectively) [42,43]. It is known that *Citrus* spp. oils contain high quantities of terpenes, especially limonene, a well-established chemopreventive and therapeutic agent against numerous tumor cells [44,45,46,47]. Our findings indicated that *C. limon* oils that contained high relative amount of limonene (33.6%) exhibited the highest cytotoxicity against HeLa cells, which agreed with a previous finding, where limonene demonstrated potential cytotoxicity (22.1 μg/mL) against HeLa cells [48]. Surprisingly, *C. pyriformis,* which contained the highest proportion of limonene (70.4%), displayed the lowest antiproliferative activity. A few studies have highlighted that limonene might not be a primary contributor to the cytotoxicity of essential oils [49,50,51]. On the other hand, several other terpenes that are found in *Citrus* spp. oils were reported to exhibit a better cytotoxic potential against HeLa cells. For instance, citral demonstrate the ability to suppress cell proliferation, through the increment of intracellular reactive oxygen species and dissipation of mitochondrial membrane potential in HeLa cells [52]. Linalool can induce apoptosis in HeLa cells through cyclin-dependent kinase inhibitors and src kinases [53]. Other compounds with a cytotoxicity effect against various cancer cell lines include phytol, nerolidol, α-humulene, geraniol, β-caryophyllene, and β-elemene [54,55,56,57,58,59]. These findings suggest that different constituents in *Citrus* EOs may synergistically contribute to their antiproliferative activity against HeLa cells, instead of being the sole contribution of a single bioactive compound.

## 3. Materials and Methods

### 3.1. Chemicals and Reagents

HPLC-grade methanol, acetone, and hexane were purchased from Elite Advanced Materials Sdn. Bhd. (Selangor, Malaysia). Ethanol was purchased from Merck (Darmstadt, Germany). High-purity (≥99%) *n*-alkanes (heptane, octane, nonane, decane, undecane, dodecane, tridecane, tetradecane, pentadecane, hexadecane, heptadecane, octadecane, nonadecane, eicosane, heneicosane, docosane, tricosane, tetracosane, pentacosane, hexacosane, octacosane, and triacontane) and 2,2-diphenylpicrylhydrazyl (DPPH) were purchased from Sigma-Aldrich (St. Louis, MO, USA). Ultra-pure water (18.2 MΩ cm^−1^) was produced using a Millipore Milli-Q ultrapure water purification system (Bedford, MA, USA).

### 3.2. Plant Material and Isolation of Essential Oil

*Citrus* spp. leaf samples were collected from selected orchards located at Gemencheh, Negeri Sembilan—*C. hystrix* and *C. limon*; Batu Ferringhi, Penang—*C. pyriformis*; and Gelugor, Penang—*C. microcarpa* in March 2021. Approximately 400 g of fresh leaves was subjected to steam distillation (Figure S1) for 3 h. The yields for the steam-distilled *Citrus* spp. leaf EOs ranged from 0.28 to 0.72% (*w*/*w*) on fresh weight basis (Table S2; Supplementary Information). The essential oil layer was collected and stored at 4 °C in glass vials, until further being analyzed, and were then diluted in 2% *v/v* acetone prior to being injected into the GC system.

### 3.3. Gas Chromatography–Quadrupole Mass Spectrometry System

Chemical profiling of the *Citrus* spp. leaf EOs was conducted on an Agilent Technologies 7890B gas chromatography system (Agilent Technologies, Santa Clara, CA, USA) equipped with a 5977B GC/MSD quadrupole mass spectrometer, 7693A autosampler, and a split/splitless inlet. The separation was effected using a nonpolar (HP-5ms (5% phenyl-methylpolysiloxane)) capillary column of dimensions 30 m × 0.25 mm I.D. × 0.25 μm *d*_f_. The GC conditions used were oven temperature program, 40 °C (hold 2 min) at 3 °C min^−1^ to 220 °C, 2 °C min^−1^ to 300 °C; injector temperature of 250 °C; helium (purity of 99.999%) at a flow rate of 1.0 mL min^−1^; and injection volume of 1 μL, and using a split ratio of 5:1. The quadrupole MS was operated in 70 eV electron ionization mode, ion source temperature of 230 °C, solvent delay time of 3.0 min, deactivated fused-silica capillary as the transfer line (0.8 m × 0.1 mm I.D.) thermostated at 280 °C, and a mass scan range of 45–600 Da.

### 3.4. Data Handling

GC-QMS data acquisition and processing were performed using Agilent Mass Hunter Qualitative Analysis 10.0 (Agilent Technologies). The chemical constituents of the EOs were tentatively identified using their retention indices (RI) (relative to n-alkanes C_7_–C_30_), which were calculated using the Van den Dool and Kratz equation and mass spectrum matching, according to the NIST 14.0 MS library database (National Institute of Standards and Technology; Gaithersburg, MD, USA), and compared with previously reported RI values [60,61]. A matching score ≥ 80 in conjunction with consistent RI values within ±10 when compared to reported RI, were employed as criteria for the tentative identification of the chromatographically separated compounds. The relative concentration of tentatively identified compounds was calculated based on the acquired TICs and presented as the mean ± standard deviation from three repeated samples injections. Principal component analysis (PCA) was performed using Unscrambler X 10.3 (CAMO Software, Oslo, Norway) to identify differences for the obtained chemical profiles. All data were presented using Excel software (Microsoft Corporation, Washington, DC, USA) and Origin 8 (Origin Lab Corporation, Northampton, MA, USA). One-way analysis of variance (ANOVA) was performed using MS Excel software (Version 2211 Build 16.0.15831.20098).

### 3.5. Antioxidant Activity by DPPH Assay

The scavenging activity of four *Citrus* spp. leaf essential oils was determined using the DPPH method, as reported by Gursoy et al. [62], with slight modifications. Briefly, *C. limon*, *C. pyriformis*, and *C. microcarpa* EOs were prepared in methanol at different concentrations (3.12, 6.25, 12.50, 25.00, 50.00, and 100 mg/mL), while *C. hystrix* was prepared in a concentration range of 3.12–400 mg/mL. Subsequently, 50 µL of oil was added to 150 µL of the methanolic DPPH solution (0.1 mM), incubated in 96-well microplate for 30 min, and protected from light. Ascorbic acid solutions in methanol were prepared and used as a positive reference standard; as a negative control, methanol solution was used. At the end of the incubation period, the absorbance was immediately measured at 517 nm using an Epoch microplate UV-Vis spectrophotometer (BioTek Instruments Inc., Winooski, VT, USA). The assay was conducted in triplicate. The DPPH free-radical scavenging ability was calculated using the following equation:

(1) $$\mathrm{DPPH}\;\mathrm{scavenging}\;\mathrm{activity}\;(\%)=(\frac{{\mathrm{Abs}}_{\mathrm{control}}-{\mathrm{Abs}}_{\mathrm{sample}}}{{\mathrm{Abs}}_{\mathrm{control}}})\times \mathrm{100}$$

### 3.6. HeLa Cell Culture

The HeLa cell line was purchased from the American Type Culture Collection (Manassas, VA, USA). The HeLa cells were cultured in Dulbecco’s modified Eagle’s medium—high glucose (DMEM; Sigma-Aldrich), with 10% fetal bovine serum and 1% penicillin or streptomycin at a temperature of 37 °C and with 5% CO_2_.

### 3.7. Antiproliferative Activity with 3-(4,5-Dimethylthiazol-2-yl)-2,5-diphenyltetrazolium Bromide (MTT) Assay

Cells were seeded in 96 well plates at a density of 3000 cells/well supplemented DMEM (Dulbecco’s modified Eagle’s medium) and incubated for 24 h in a humified atmosphere (37 °C and 5% CO_2_). *Citrus* leaf EOs were re-dissolved in ethanol at a final concentration of 50 mg/mL, and the medium was removed, and each well was supplemented with different concentrations of EOs (2.34 to 150.00 μg/mL) diluted with DMEM. The ethanol concentration in each well was ≤0.3%. An MTT assay was conducted after the 24 h incubation period, to determine cell viability via the measurement of color alterations, which gauge the activity of the enzyme that reduces MTT to formazan, giving a purple color [54]. The plates were measured for optical density at 540 nm using a SkanIT absorbance micro-plate reader (Thermo Scientific, St. Peters, MO, USA). Media containing cells were used as a positive control, while media containing no cells were used as a negative control. Cells and medium with 0.3% ethanol were used as the vehicle control. All the assays were conducted in triplicate. The percentage of cell viability was determined using the following equation:

(2) $$\mathrm{Cell}\;\mathrm{viability}\;(\%)=(\frac{\mathrm{Mean}\;\mathrm{absorbance}\;\mathrm{in}\;\mathrm{test}\;\mathrm{well}}{\mathrm{Mean}\;\mathrm{absorbance}\;\mathrm{in}\;\mathrm{vehicle}\;\mathrm{control}\;\mathrm{well}})\times \mathrm{100}$$

## 4. Conclusions

The present study detailed the metabolic profiling of secondary compounds in leaf essential oils of *C. hystrix*, *C. limon*, *C. pyriformis*, and *C. microcarpa* sourced in Malaysia. The metabolic profiles revealed notable differences for phytoconstituents within the four *Citrus* spp. leaf oils. *C. hystrix* was dominated by oxygenated monoterpenes (89.5%), *C. microcarpa* was dominated by oxygenated sesquiterpenes (38.9%), and *C. pyriformis* and *C. limon* were dominated by monoterpene hydrocarbons (≥48.6%). PCA specifically revealed the discriminating metabolites and allowed chemotaxonomical classification according to *Citrus* species. All EOs showed DPPH radical scavenging ability, with *C. limon* and *C. pyriformis* exhibiting higher antioxidant activities (IC_50_ < 40 mg/mL). Additionally, all the analyzed oils displayed potent antiproliferative activities against the HeLa cell line, with *C. limon* showing the highest antiproliferative activity, with an IC_50_ value of 11.7 μg/mL. The discrepancies observed in the antioxidant and antiproliferative activities of the leaf oils extracted from different *Citrus* spp. suggest an inter-relation of metabolic diversities in effecting these bioactivities via specific molecular mechanisms, which will require further investigations. Nevertheless, this study can serve as an a priori reference for the development of bioactive foods and nutraceuticals incorporated with *Citrus* leaf oil that can provide health-promoting properties.

## Figures and Tables

**Table 1:** Major secondary compounds tentatively identified in *Citrus* spp. leaf EOs using GC-MS.

No	Compounds	CASRN	Molecular Formula	Molecular Weight	^a^ Class	^b^ Match Factor (Reverse Match Factor)	RI_ref_	^c^*m*/*z* of Significant Ions (Relative Ion Abundance)	^d^ RI_cal_			
									^e^ (Relative Percentage Abundance, %)			
									CL	CH	CM	CP
1	Pinene, α-	80-56-8	C_10_H_16_	136.13	*MH*	948 (949); 940 (943); 952 (954); 945 (948)	935	93.1 (100), 91.1 (48.75), 92.1 (39.17); 93.1 (100), 91.1 (48.10), 92.1 (36.00); 93.1 (100), 91.1 (49.53), 92.1 (38.79); 93.1 (100), 91.1 (47.73), 92.1 (38.09)	930 (0.67 ± 0.01)	930 (0.14 ± 0.01)	931 (1.41 ± 0.03)	930 (0.60 ± 0.01)
2	Sabinene	3387-41-5	C_10_H_16_	136.13	*MH*	941 (948); 949 (955); 949 (957)	971	93.1 (100), 91.1 (50.15), 77.1 (38.85); 93.1 (100), 91.1 (51.67), 77.1 (39.52); 93.1 (100), 91.1 (49.63), 77.1 (38.47)	971 (0.69 ± 0.03)	972 3.02 ± 0.06)	NA	971 0.89 ± 0.01)
3	Pinene, β-	18172-67-3	C_10_H_16_	136.13	*MH*	948 (948); 936 (936); 941 (941); 940 (943)	973	93.1 (100), 91.1 (33.03), 79.1 (26.68); 93.1 (100), 91.1 (33.43), 79.1 (26.28); 93.1 (100), 91.1 (33.39), 79.1 (26.96); 93.1 (100), 91.1 (34.28), 79.1 (25.15)	973 (1.83 ± 0.02)	973 (0.16 ± 0.05)	978 (7.12 ± 0.23)	973 (0.12 ± 0.02)
4	Myrcene, β-	123-35-3	C_10_H_16_	136.13	*MH*	939 (953); 949 (964); 940 (956); 949 (962)	991	93.1 (100), 69.1 (61.56), 91.1 (27.58); 93.1 (100), 69.1 (60.67), 91.1 (27.67); 93.1 (100), 69.1 (60.51), 91.1 (27.26); 93.1 (100), 69.1 (60.26), 91.1 (27.74)	992 (1.78 ± 0.02)	991 (0.80 ± 0.01)	991 (0.28 ± 0.01)	992 (1.63 ± 0.01)
5	3-Carene	13466-78-9	C_10_H_16_	136.13	*MH*	934 (934); 926 (933); 904 (921); 897 (906)	1008	93.1 (100), 91.1 (56.18), 77.1 (35.85); 93.1 (100), 91.1 (55.54), 77.1 (44.27); 93.1 (100), 91.1 (51.84), 77.1 (33.96); 93.1 (100), 91.1 (47.73), 77.1 (35.68)	1010 (5.42 ± 0.08)	1008 (0.05 ± 0.01)	1008 (0.38 ± 0.01)	1008 (0.03 ± 0.01)
6	Limonene	138-86-3	C_10_H_16_	136.13	*MH*	952 (953); 905 (905); 935 (936); 955 (956)	1033	93.1 (100), 68.2 (98.59), 67.2 (82.02); 93.1 (100), 68.2 (62.14), 67.1 (53.19); 93.1 (100), 68.1 (73.71), 67.2 (63.04); 93.2 (99.09), 68.2 (100), 67.2 (80.60)	1033 (33.57 ± 0.54)	1026 (0.21 ± 0.02)	1027 (1.70 ± 0.03)	1038 (70.40 ± 0.46)
7	Ocimene, β-	13877-91-3	C_10_H_16_	136.13	*MH*	946 (946); 939 (939); 952 (952); 945 (945)	1050	93.1 (100), 91.1 (56.62), 79.1 (46.19); 93.1 (100), 91.1 (53.24), 79.1 (44.57); 93.1 (100), 91.1 (53.72), 79.1 (44.23); 93.1 (100), 91.1 (55.17), 79.1 (44.64)	1050 (1.96 ± 0.02)	1048 (0.48 ± 0.01)	1050 (2.36 ± 0.03)	1051 (1.91 ± 0.01)
8	Terpinolene	586-62-9	C_10_H_16_	136.13	*MH*	926 (942); 919 (922); 939 (944); 922 (925)	1087	121.1 (100), 93.1 (99.35), 136.2 (87.49); 121.1 (100), 136.1 (93.82), 93.1 (89.14); 121.1 (100), 93.1 (99.73), 136.1 (91.90); 121.1 (100), 93.1 (95.15), 136.2 (82.49)	1087 (1.54 ± 0.02)	1086 (0.13 ± 0.01)	1086 (0.13 ± 0.01)	1087 (0.08 ± 0.01)
9	Linalool	78-70-6	C_10_H_18_O	154.14	*OM*	932 (932); 912 (913); 946 (946); 946 (946)	1101	71.1 (100), 93.1 (97.92), 55.2 (55.40); 71.1 (100), 93.1 (98.28), 55.1 (54.53); 71.1 (100), 93.1 (98.79), 55.1 (53.45); 71.1 (100), 93.1 (97.83), 55.1 (55.05)	1100 (0.73 ± 0.01)	1101 (3.20 ± 0.01)	1103 (2.90 ± 0.04)	1101 (1.24 ± 0.01)
10	Citronellal	106-23-0	C_10_H_18_O	154.14	*MA*	925 (925); 920 (931); 909 (909); 923 (923)	1157	69.2 (100), 95.1 (83.95), 55.1 (47.81); 69.2 (100), 95.1 (85.86), 121.2 (48.93); 69.1 (100), 95.1 (84.22), 121.1 (47.39); 69.2 (100), 95.1 (87.03), 121.1 (48.31)	1155 (1.54 ± 0.01)	1169 (77.69 ± 0.37)	1154 (0.28 ± 0.01)	1157 (5.64 ± 0.02)
11	Isogeranial	55722-59-3	C_10_H_16_O	152.12	*MA*	937 (959) 931 (932)	1184	81.1 (100), 67.1 (84.07), 109.1 (76.29); 81.1 (100), 67.1 (84.18), 109.1 (74.65)	1184 (1.20 ± 0.01)	NA	NA	1184 (0.22 ± 0.01)
12	Citronellol, β-	106-22-9	C_10_H_20_O	156.15	*OM*	937 (937); 936 (937); 905 (905); 948 (950)	1236	69.1 (100), 67.1 (47.78), 81.1 (36.60); 69.1 (100), 67.1 (71.79), 81.1 (66.00); 69.1 (100), 67.1 (56.05), 81.2 (59.75); 69.1 (100), 67.1 (70.83), 81.1 (65.03)	1236 (5.89 ± 0.04)	1233 (3.75 ± 0.06)	1229 (0.02 ± 0.01)	1231 (0.64 ± 0.01)
13	Citral, β-	106-26-3	C_10_H_16_O	152.12	*MA*	949 (950); 942 (943)	1242	69.2 (100), 109.1 (48.27), 94.1 (38.81); 69.2 (100), 109.1 (45.21), 94.1 (37.45)	1247 (9.11 ± 0.04)	NA	NA	1243 (1.60 ± 0.01)
14	Citral, α-	141-27-5	C_10_H_16_O	152.12	*MA*	949 (949); 942 (942)	1287	69.2 (100), 84.1 (27.65), 94.1 (19.55); 69.2 (100), 84.1 (28.80), 94.1 (20.16)	1280 (12.02 ± 0.07)	NA	NA	1275 (2.03 ± 0.02)
15	EIemene, δ-	20307-84-0	C_15_H_24_	204.19	*SH*	942 (950); 943 (951); 913 (917)	1338	121.1 (100), 93.1 (53.09), 107.1 (41.12); 121.1 (100), 93.1 (65.01), 136.2 (58.35); 121.2 (100), 93.1 (67.04), 136.2 (58.88)	NA	1336 (0.03 ± 0.01)	1338 (3.22 ± 0.01)	1337 (0.11 ± 0.01)
16	Citronellol acetate	150-84-5	C_12_H_22_O_2_	198.17	*MAc*	954 (954); 950 (950); 872 (881); 908 (908)	1355	81.1 (100), 95.1 (97.44), 69.1 (90.52); 95.1 (100), 81.1 (98.43), 69.1 (83.91); 81.1 (100), 95.1 (92.44), 69.1 (87.26); 81.1 (100), 95.1 (96.81), 69.1 (94.12)	1355 (0.14 ± 0.03)	1357 (2.81 ± 0.06)	1355 (0.02 ± 0.01)	1355 (0.19 ± 0.01)
17	Nerol acetate	141-12-8	C_12_H_20_O_2_	196.15	*MAc*	934 (935); 906 (908); 907 (907); 932 (932)	1367	69.2 (100), 93.1 (55.87), 68.2 (38.06); 69.2 (100), 93.2 (53.09), 68.1 (34.91); 69.2 (100), 93.2 (59.57), 68.1 (38.53); 69.1 (100), 93.1 (61.17), 68.1 (41.17)	1369 (3.57 ± 0.10)	1366 (0.25 ± 0.03)	1366 (0.03 ± 0.01)	1367 (0.73 ± 0.01)
18	Geranyl acetate	16409-44-2	C_12_H_20_O_2_	196.15	*MAc*	951 (958); 916 (926); 914 (914)	1386	69.2 (100), 68.2 (36.97), 93.1 (34.17); 69.1 (100), 68.2 (38.07), 93.1 (35.94); 69.2 (100), 68.2 (36.12), 93.1 (35.08)	1376 (2.92 ± 0.04)	1386 (0.96 ± 0.02)	NA	1384 (0.39 ± 0.03)
19	Elemene, β-	515-13-9	C_15_H_24_	204.19	*SH*	918 (918); 919 (921); 929 (931)	1393	93.1 (100), 81.1 (86.08), 67.1 (83.02); 93.1 (100), 81.1 (82.01), 107.1 (72.88); 93.1 (100), 81.1 (82.01), 107.1 (74.12)	NA	1391 (0.05 ± 0.01)	1392 (1.32 ± 0.01)	1392 (2.78 ± 0.08)
20	Caryophyllene	87-44-5	C_15_H_24_	204.19	*SH*	923 (923); 952 (952); 937 (937); 950 (950)	1418	91.1 (100), 133.1 (94.72), 93.1 (83.53); 133.1 (100), 91.1 (92.12), 93.1 (87.47); 133.1 (100), 91.1 (95.02), 93.1 (82.61); 133.1 (100), 91.1 (91.34), 93.1 (85.56)	1418 (1.48 ± 0.02)	1418 (0.45 ± 0.01)	1419 (3.29 ± 0.01)	1418 (1.64 ± 0.03)
21	Germacrene D	23986-74-5	C_15_H_24_	204.19	*SH*	888 (902); 946 (959); 922 (936)	1480	161.2 (100), 105.1 (50.92), 91.1 (47.97); 161.2 (100), 105.1 (49.83), 91.1 (47.17); 161.2 (100), 105.1 (49.62), 91.1 (47.63)	NA	1479 (0.04 ± 0.01)	1486 (13.04 ± 0.25)	1479 (0.36 ± 0.01)
22	Selinene, β-	17066-67-0	C_15_H_24_	204.19	*SH*	948 (958); 931 (935)	1489	105.1 (100), 93.2 (93.51), 107.1 (89.08); 93.2 (100), 105.1 (97.15), 107.1 (84.76)	NA	NA	1489 (2.42 ± 0.17)	1484 (0.10 ± 0.01)
23	Bicyclogermacrene	24703-35-3	C_15_H_24_	204.19	*SH*	920 (921); 920 (920); 923 (924); 918 (918)	1495	121.2 (100), 93.1 (65.23), 107.1 (48.60); 121.2 (100), 93.1 (67.06), 107.1 (51.53); 121.1 (100), 93.1 (67.78), 107.1 (51.70); 121.1 (100), 93.1 (86.30), 107.1 (52.65)	1495 (0.13 ± 0.01)	1495 (0.33 ± 0.17)	1497 (2.03 ± 0.38)	1495 (0.14 ± 0.01)
24	Elemol	639-99-6	C_15_H_26_O	222.19	*OS*	943 (950); 950 (958); 933 (940)	1549	93.1 (100), 161.2 (97.07), 59.1 (93.10); 93.1 (100), 161.2 (94.41), 59.1 (82.73); 93.1 (100), 161.2 (89.99), 59.1 (88.31)	NA	1548 (0.42 ± 0.05)	1557 (16.67 ± 0.10)	1548 (0.19 ± 0.01)
25	Nerolidol	40716-66-3	C_15_H_26_O	222.19	*OS*	863 (863); 931 (938); 941 (950); 906 (911)	1564	69.2 (100), 93.1 (84.83), 107.1 (51.52); 69.2 (100), 93.1 (97.66), 107.1 (66.85); 69.2 (100), 93.1 (93.98), 107.1 (64.33); 69.2 (100), 93.1 (96.15), 107.2 (66.52)	1564 (0.02 ± 0.01)	1564 (0.80 ± 0.10)	1567 (1.53 ± 0.01)	1564 (0.09 ± 0.01)
26	Eudesmol, epi-γ-	117066-77-0	C_15_H_26_O	222.19	*OS*	933 (951)	1620	189.2 (100), 162.1 (72.76), 204.2 (61.03)	NA	NA	1520 (1.70 ± 0.04)	NA
27	Eudesmol, γ-	1209-71-8	C_15_H_26_O	222.19	*OS*	932 (933)	1635	189.2 (100), 161.2 (95.46), 204.2 (78.66)	NA	NA	1635 (5.69 ± 0.20)	NA
28	Eudesmol, β-	473-15-4	C_15_H_26_O	222.19	*OS*	952 (960)	1656	149.2 (100), 59.2 (78.76), 164.2 (44.78)	NA	NA	1656 (8.58 ± 0.05)	NA
29	Eudesmol, α-	473-16-5	C_15_H_26_O	222.19	*OS*	940 (951)	1659	149.2 (100), 161.2 (98.27), 204.2 (87.13)	NA	NA	1659 (3.62 ± 0.04)	NA
30	Phytol	150-86-7	C_20_H_40_O	296.31	*OD*	929 (931); 911 (913); 922 (923); 929 (931)	2113	71.1 (100), 123.2 (43.38), 81.1 (33.37); 71.1 (100), 123.1 (44.94), 57.1 (38.08); 71.1 (100), 123.1 (44.15), 81.2 (38.00); 71.1 (100), 123.2 (47.34), 81.2 (39.51)	2113 (1.62 ± 0.12)	2113 (0.21 ± 0.02)	2112 (0.39 ± 0.01)	2113 (0.78 ± 0.04)

^a^ Class of chemical compounds: *MH* monoterpenic hydrocarbon, *MA* monoterpenic aldehyde, *OM* monoterpenic alcohol, *MAc* monoterpenic acetate, *SH* sesquiterpenic hydrocarbon, *OS* sesquiterpenic alcohol, *OD* oxygenated diterpene. ^b^ Matching scores of compounds reported at 80%, based on the mass spectra in NIST library database and in the order *C. limon* (CL)*, C. hystrix* (CH), *C. microcarpa* (CM), and *C. pyriformis* (CP). ^c^ Fragmentation patterns reported in order *C. limon*, *C. hystrix*, *C. microcarpa*, and *C. pyriformis*. ^d^ Retention index (RI) values calculated using the Van Den Dool and Kratz equation with reference to the reported RI values within the range of ±10. ^e^ Relative percentage abundance calculated on the basis of the TIC area, as the percentage of the total TIC area.

**Table 2:** Antioxidant activity of *Citrus* spp. leaf essential oils.

*Citrus* spp.	IC_50_ (mg/mL)	IC_50_ (mg/mL) *	References
*C. hystrix*	279.03 ± 10.34	>0.25	[36]
*C. limon*	29.14 ± 1.97	6.47	[37]
*C. pyriformis*	39.99 ± 0.73	28.91	[38]
*C. microcarpa*	59.42 ± 1.77	~0.05	[13]
Ascorbic acid (control)	0.43 ± 1.70	0.42	[39]

* Values reported from previous studies.

## Data Availability

Not applicable.

## Supplementary Materials

The following supporting information can be downloaded at: https://www.mdpi.com/article/10.3390/plants12010134/s1, Figure S1. Steam distillation equipment used for the extraction of *Citrus* spp. leaf essential oils. Table S1. Secondary compounds identified in different *Citrus* spp. leaf oils using GC-MS. Table S2. Yields of four *Citrus* spp. leaf essential oils.
