# Supplementary material for: Phytochemical Composition, Antioxidant and Antiproliferative Activities of Citrus hystrix, Citrus limon, Citrus pyriformis, and Citrus microcarpa Leaf Essential Oils against Human Cervical Cancer Cell Line
Source: Plants (Basel). 2022 Dec 27;12(1):134. doi: 10.3390/plants12010134 (PMC9823843; doi:10.3390/plants12010134)
Supplement: Supplementary file 1 [file plants-12-00134-s001.zip › plants-2064451-supplementary.pdf]

Phytochemical Composition, Antioxidant and Antiproliferative Activities of *Citrus hystrix*, *Citrus limon*, *Citrus pyriformis*, and *Citrus microcarpa* Leaf Essential Oils against Human Cervical Cancer Cell Line

Haneen Ibrahim Al Othman [1], Huda Hisham Alkatib [2], Atiqah Zaid [1], Sreenivasan Sasidharan [2], Siti Sarah Fazalul Rahiman [3], Tien Ping Lee [4,*], George Dimitrovski [5], Jalal T. Althakafy [6] and Yong Foo Wong [1,*]

1. Centre for Research on Multidimensional Separation Science, School of Chemical Sciences, Universiti Sains Malaysia, Penang 11800, Malaysia
2. Institute for Research in Molecular Medicine (INFORMM), Universiti Sains Malaysia, Penang 11800, Malaysia
3. School of Pharmaceutical Sciences, Universiti Sains Malaysia, Penang 11800, Malaysia
4. RCSI & UCD Malaysia Campus, 4 Jalan Sepoy Lines, Penang 10450, Malaysia
5. Ajoya Capital Limited, World Trade Centre 1, Jl. Jenderal Sudirman Kav. 29-31, Jakarta 12920, Indonesia
6. Department of Chemistry, Faculty of Applied Science, Umm Al-Qura University, Makkah 21955, Saudi Arabia

* Correspondence: leetienping@rcsiucd.edu.my (T.P.L.); wongyongfoo@usm.my (Y.F.W.);
Tel.: +60-4653-4031 (Y.F.W.)

Supplementary material

Table S1. Secondary compounds identified in different *Citrus* spp. leaf oils using GC–MS.

No	Compounds	CASRN	Molecular formula	[a]Class	[b]Match Factor (Reverse Match Factor)	RI_{ref}	[c]m/z of significant ions (relative ion abundance)	[d]RIcal [e](Relative percentage abundance, %)			
								CL	CH	CM	CP
1	Thujene, α-	5-2-2867	$C_{10}H_{16}$	*MH*	918 (918); 917 (918); 932 (947); 920 (920)	925	93.1 (100), 91.1 (74.56), 77.1 (42.36); 93.1 (100), 91.1 (65.57), 77.1 (40.07); 93.1 (100), 91.1 (65.97), 77.1 (42.24); 93.1 (100), 91.1 (65.93), 77.1 (39.69)	925 (0.03± 0.01)	925 (0.03± 0.01)	925 (0.13± 0.01)	925 (0.04± 0.01)
2	Pinene, α-	80-56-8	$C_{10}H_{16}$	*MH*	948 (949); 940 (943); 952 (954); 945 (948)	935	93.1 (100), 91.1 (48.75), 92.1 (39.17); 93.1 (100), 91.1 (48.10), 92.1 (36.00); 93.1 (100), 91.1 (49.53), 92.1 (38.79); 93.1 (100), 91.1 (47.73), 92.1 (38.09)	930 (0.67± 0.01)	930 (0.14± 0.01)	931 (1.41± 0.03)	930 (0.60± 0.01)
3	Camphene	79-92-5	$C_{10}H_{16}$	*MH*	901 (901); 854 (854); 944 (957); 877 (877)	944	93.1 (100), 121.2 (74.53), 91.0 (53.75); 93.1 (100), 121.2 (57.71), 76.9 (62.38); 93.1 (100), 121.1 (73.25), 91.1 (37.15); 93.1 (100), 121.0 (86.33), 77.1 (44.80)	944 (0.02± 0.01)	944 (0.01± 0.01)	944 (0.100± 0.01)	944 (0.01± 0.01)
4	Sabinene	3387-41-5	$C_{10}H_{16}$	*MH*	941 (948); 949 (955); 949 (957)	971	93.1 (100), 91.1 (50.15), 77.1 (38.85); 93.1 (100), 91.1 (51.67), 77.1 (39.52); 93.1 (100), 91.1 (49.63), 77.1 (38.47)	971 (0.69± 0.03)	972 3.02± 0.06)	NA	971 0.89± 0.01)
5	Pinene, β-	18172-67-3	$C_{10}H_{16}$	*MH*	948 (948); 936 (936); 941 (941); 940 (943)	973	93.1 (100), 91.1 (33.03), 79.1 (26.68); 93.1 (100), 91.1 (33.43), 79.1 (26.28); 93.1 (100), 91.1 (33.39), 79.1 (26.96); 93.1 (100), 91.1 (34.28), 79.1 (25.15)	973 (1.83± 0.02)	973 (0.16± 0.05)	978 (7.12± 0.23)	973 (0.12± 0.02)
6	Myrcene, β-	123-35-3	$C_{10}H_{16}$	*MH*	939 (953); 949 (964); 940 (956); 949 (962)	991	93.1 (100), 69.1 (61.56), 91.1 (27.58); 93.1 (100), 69.1 (60.67), 91.1 (27.67); 93.1 (100), 69.1 (60.51), 91.1 (27.26); 93.1 (100), 69.1 (60.26), 91.1 (27.74)	992 (1.78± 0.02)	991 (0.80± 0.01)	991 (0.28± 0.01)	992 (1.63± 0.01)
7	Phellandren, α-	99-83-2	$C_{10}H_{16}$	*MH*	926 (927); 908 (908); 923 (925);	1002	93.1 (100), 91.1 (64.51), 77.1 (39.28); 93.1 (100), 91.1 (77.61), 77.1 (38.79); 93.1 (100), 91.1 (64.64), 77.1 (37.23)	1003 (0.48± 0.01)	1002 (0.02± 0.01)	1002 (0.14± 0.01)	NA

8	3-Carene	13466-78-9	$C_{10}H_{16}$	*MH*	934 (934); 926 (933); 904 (921); 897 (906)	1008	93.1 (100), 91.1 (56.18), 77.1 (35.85); 93.1 (100), 91.1 (55.54), 77.1 (44.27); 93.1 (100), 91.1 (51.84), 77.1 (33.96); 93.1 (100), 91.1 (47.73), 77.1 (35.68)	1010 (5.42± 0.08)	1008 (0.05± 0.01)	1008 (0.38± 0.01)	1008 (0.03± 0.01)
9	Terpinene, α-	99-86-5	$C_{10}H_{16}$	*MH*	932 (940); 940 (945); 977 (935); 858 (585)	1015	121.1 (100), 93.1 (71.74), 136.1 (46.36); 121.1 (100), 93.1 (72.90), 136.1 (54.12); 121.1 (100), 93.1 (77.03), 136.1 (50.85); 121.1 (100), 93.1 (71.27), 136.1 (46.63)	1012 (0.15± 0.04)	1015 (0.05± 0.01)	1015 (0.20± 0.01)	1023 (0.02± 0.01)
10	Limonene	138-86-3	$C_{10}H_{16}$	*MH*	952 (953); 905 (905); 935 (936); 955 (956)	1033	93.1 (100), 68.2 (98.59), 67.2 (82.02); 93.1 (100), 68.2 (62.14), 67.1 (53.19); 93.1 (100), 68.1 (73.71), 67.2 (63.04); 68.2 (100), 93.2 (99.09), 67.2 (80.60)	1033 (33.57± 0.54)	1026 (0.21± 0.02)	1027 (1.70± 0.03)	1038 (70.40± 0.46)
11	Eucalyptol	470-82-6	$C_{10}H_{18}O$	*MO*	875 (875); 940 (941)	1029	81.1 (100), 108.1 (85.20), 111.1 (82.94); 81.1 (100), 108.1 (97.85), 111.1 (86.57)	NA	1029 (0.08± 0.01)	1029 (0.23± 0.01)	NA
12	Ocimene, trans-β-	3779-61-1	$C_{10}H_{16}$	*MH*	947 (947); 917 (917); 949 (950); 936 (939)	1038	93.1 (100), 91.1 (53.07), 79.1 (40.33); 93.1 (100), 91.1 (51.45), 79.1 (43.22); 93.1 (100), 91.1 (50.91), 92.1 (39.45); 93.1 (100), 91.1 (53.60), 92.1 (39.22)	1039 (0.34± 0.01)	1038 (0.044± 0.01)	1038 (0.11± 0.01)	1038 (0.16± 0.02)
13	Ocimene, β-	13877-91-3	$C_{10}H_{16}$	*MH*	951 (952); 934 (934); 949 (949); 945 (945)	1050	93.1 (100), 91.1 (56.62), 79.1 (46.19); 93.1 (100), 91.1 (53.24), 79.1 (44.57); 93.1 (100), 91.1 (53.72), 79.1 (44.23); 93.1 (100), 91.1 (55.17), 79.1 (44.64)	1050 (1.96± 0.02)	1048 (0.48± 0.01)	1050 (2.36± 0.03)	1051 (1.91± 0.01)
14	Terpinene, γ-	99-85-4	$C_{10}H_{16}$	*MH*	955 (956); 950 (950); 947 (953); 955 (956)	1057	93.1 (100), 91.1 (65.90), 136.1 (45.44); 93.1 (100), 91.1 (64.25), 136.1 (47.04); 93.1 (100), 91.1 (57.87), 136.1 (44.36); 93.1 (100), 91.1 (64.92), 136.1 (45.81)	1058 (0.15± 0.03)	1057 (0.08± 0.01)	1057 (0.29± 0.01)	1058 (0.25± 0.01)
15	4-Thujanol	15537-55-0	$C_{10}H_{18}O$	*OM*	842 (842); 923 (923); 852 (852); 823 (827)	1060	93.1 (100), 71.1 (95.60), 111.1 (79.24); 93.1 (100), 71.1 (93.20), 111.1 (80.16); 71.1 (100), 93.1 (81.98), 111.1 (76.47); 93.1(100), 71.1 (69.43), 111.1 (68.32)	1065 (0.01± 0.01	1065 (0.05± 0.01)	1065 (0.01± 0.01)	1066 (0.01± 0.01)

16	1-Octanol	111-87-5	$C_8H_{18}O$	*OA*	926 (926); 862 (878); 921 (933)	1072	56.1 (100), 55.1 (95.02), 70.0 (82.33); 56.2 (100), 55.1 (69.86), 69.1 (65.09); 56.2 (100), 55.1 (90.41), 69.2 (80.08)	1072 (0.01± 0.01)	NA	1072 (0.01± 0.01)	1072 (0.01± 0.01)
17	Trans-linalool oxide	34995-77-2	$C_{10}H_{18}O_2$	*MO*	946 (949)	1071	59.1 (100), 94.2 (66.84), 93.1 (54.80)	NA	1071 (0.07± 0.01)	NA	NA
18	Terpinolene	586-62-9	$C_{10}H_{16}$	*MH*	926 (942); 919 (922); 939 (944); 922 (925)	1087	121.1 (100), 93.1 (99.35), 136.2 (87.49); 121.1 (100), 136.1 (93.82), 93.1 (89.14); 121.1 (100), 93.1 (99.73), 136.1 (91.90); 121.1 (100), 93.1 (95.15), 136.2 (82.49)	1087 (1.54± 0.02)	1086 (0.13± 0.01)	1086 (0.13± 0.01)	1087 (0.08± 0.01)
19	Linalool	78-70-6	$C_{10}H_{18}O$	*OM*	932 (932); 912 (913); 946 (946); 946 (946)	1101	71.1 (100), 93.1 (97.92), 55.2 (55.40); 71.1 (100), 93.1 (98.28), 55.1 (54.53); 71.1 (100), 93.1 (98.79), 55.1 (53.45); 71.1 (100), 93.1 (97.83), 55.1 (55.05)	1100 (0.73± 0.01)	1101 (3.20± 0.01)	1103 (2.90± 0.04)	1101 (1.24± 0.01)
20	Nonanal	124-19-6	$C_9H_{18}O$	*AA*	907 (907); 845 (866); 933 (934)	1103	57.1 (100), 56.1 (66.42), 55.2 (59.58); 57.1 (100), 56.1 (81.51), 55.2 (74.65); 57.1 (100), 56.2 (60.98), 55.1 (59.00)	1104 (0.08± 0.02)	NA	1105 (0.01± 0.01)	1105 (0.10± 0.01)
21	Cosmene	460-01-5	$C_{10}H_{14}$	*MH*	932 (938)	1129	91.1 (100), 119.1 (95.35), 134.1 (55.39)	NA	NA	1129 (0.13± 0.01)	NA
22	Isopulegol	89-79-2	$C_{10}H_{18}O$	*OM*	955 (940); 919 (919)	1145	121.1 (100), 67.1 (91.45), 81.1 (89.36); 121.1 (100), 67.1 (76.94), 93.2 (75.39)	NA	1145 (0.43± 0.03)	NA	1143 (0.03± 0.01)
23	Citronellal	106-23-0	$C_{10}H_{18}O$	*MA*	925 (925); 920 (931); 909 (909); 923 (923)	1157	69.2 (100), 95.1 (83.95), 55.1 (47.81); 69.2 (100), 95.1 (85.86), 121.2 (48.93); 69.2 (100), 95.1 (84.22), 121.1 (47.39); 69.2 (100), 95.1 (87.03), 121.1 (48.31)	1155 (1.54± 0.01)	1169 (77.69± 0.37)	1154 (0.28± 0.01)	1157 (5.64± 0.02)
24	Isoneral	1754-00-3	$C_{10}H_{16}O$	*MA*	935 (935); 920 (920); 924 (925)	1165	109.1 (100), 81.1 (94.44), 67.2 (93.65); 57.1 (100), 81.1 (95.44), 55.1 (94.30); 109.1 (100), 81.1 (99.68), 67.1 (96.25)	1166 (0.78± 0.02)	1183 (0.02± 0.01)	NA	1165 (0.16± 0.01)

25	Terpinen-4-ol	562-74-3	$C_{10}H_{18}O$	*OM*	916 (918); 907 (923); 907 (910); 905 (905)	1176	71.1 (100), 93.1 (83.14), 111.1 (74.28); 71.1 (100), 93.1 (80.96), 111.1 (79.27); 71.1 (100), 93.1 (76.04), 111.1 (75.78); 71.1 (100), 93.1 (82.52), 111.1 (71.78)	1176 (0.16± 0.04)	1180 (0.12± 0.58)	1175 (0.20± 0.01)	1176 (0.03± 0.01)
26	Isogeranial	55722-59-3	$C_{10}H_{16}O$	*MA*	937 (959); 931 (932)	1184	81.1 (100), 67.1 (84.07), 109.1 (76.29); 81.1 (100), 67.1 (84.18), 109.1 (74.65)	1184 (1.20± 0.01)	NA	NA	1184 (0.22± 0.01)
27	Terpineol, α-	98-55-5	$C_{10}H_{18}O$	*OM*	926 (926); 921 (921); 919 (919); 915 (915)	1187	121.1 (100), 59.1 (96.93), 93.1 (89.36); 59.1 (100), 93.1 (92.44), 121.1 (91.42); 59.2 (100), 93.1 (94.28), 121.1 (90.74); 121.1 (100), 93.1 (99.04), 59.1 (98.48)	1190 (0.27± 0.01)	1192 (0.03± 0.01)	1189 (0.14± 0.01)	1189 (0.15± 0.01)
28	Decanal	112-31-2	$C_{10}H_{20}O$	*MA*	872 (878); 923 (924); 944 (944)	1206	57.1 (100), 55.1 (99.41), 82.1 (71.82); 57.1 (100), 55.1 (88.69), 82.1 (67.95); 57.1 (100), 55.1 (88.89), 82.1 (68.90)	1026 (0.07± 0.01)	NA	1206 (0.07± 0.01)	1206 (0.25± 0.01)
29	Citronellol, β-	106-22-9	$C_{10}H_{20}O$	*OM*	937 (937); 936 (937); 905 (905); 948, (950)	1236	69.1 (100), 67.1 (47.78), 81.1 (36.60); 69.1 (100), 67.1 (71.79), 81.1 (66.00); 69.1 (100), 67.1 (56.05), 81.2 (59.75); 69.1 (100), 67.1 (70.83), 81.1 (65.03)	1236 (5.89± 0.04)	1233 (3.75± 0.06)	1229 (0.02± 0.01)	1231 (0.64± 0.01)
30	Citral, β-	106-26-3	$C_{10}H_{16}O$	*MA*	949 (950); 942 (943)	1242	69.2 (100), 109.1 (48.27), 94.1 (38.81); 69.2 (100), 109.1 (45.21), 94.1 (37.45)	1247 (9.11± 0.04)	NA	NA	1243 (1.60± 0.01)
31	Geraniol	106-24-1	$C_{10}H_{18}O$	*OM*	953 (953); 878 (878); 914 (914)	1254	69.2 (100), 68.2 (20.36), 93.1 (19.18); 69.2 (100), 93.1 (22.50), 68.2 (20.05); 69.2 (100), 93.1 (19.72), 68.2 (18.11)	1261 (4.20± 0.76)	1256 (0.14± 0.04)	NA	1256 (0.10± 0.01)
32	2-Decenal	3913-81-3	$C_{10}H_{18}O$	*MA*	800 (848)		55.1 (100), 70.1 (88.70), 83.1 (57.71)	NA	NA	1261 (0.01± 0.01)	NA
33	1-Decanol	112-30-1	$C_{10}H_{22}O$	*OM*	860 (860)	1272	56.2 (100), 55.0 (96.75), 70.1 (67.21)	NA	NA	1273 (0.01± 0.01)	NA
34	Citral, α-	141-27-5	$C_{10}H_{16}O$	*MA*	949 (949); 942 (942)	1287	69.2 (100), 84.1 (27.65), 94.1 (19.55); 69.2 (100), 84.1 (28.80), 94.1 (20.16)	1280 (12.02±	NA	NA	1275 (2.03±

								0.07)			0.02)
35	Thymol	89-83-8	$C_{10}H_{14}O$	*OM*	914 (914)	1293	135.1 (100), 150.2 (29.18), 91.1 (17.81)	NA	NA	NA	1293<br>(0.12±<br>0.01)
36	Undecanal	112-44-7	$C_{11}H_{22}O$	*AA*	925 (925);<br>940 (948)	1307	57.1 (100), 55.1 (97.03), 82.2 (81.80);<br>57.1 (100), 55.1 (92.89), 82.2 (92.87)	1307<br>(0.09±<br>0.01)	NA	NA	1307<br>0.08±<br>0.01)
37	p-<br>Vinylguaiac<br>ol	7786-61-<br>0	$C_9H_{10}O_2$	*OA*	896 (906);<br>904 (904);<br>924 (925);<br>931 (933)	1312	150.1 (100), 135.1 (87.76), 107.1 (48.11);<br>150.1 (100), 135.1 (80.85), 107.1 (41.10);<br>150.1 (100), 135.1 (82.42), 107.1 (38.07);<br>150.1 (100), 135.1 (89.89), 107.1 (41.80)	1313<br>(0.08±<br>0.02)	1312<br>(0.02±<br>0.01)	1312<br>(0.06±<br>0.01)	1312<br>(0.26±<br>0.01)
38	EIemene, δ-	20307-<br>84-0	$C_{15}H_{24}$	*SH*	942 (950);<br>943 (951);<br>913 (917)	1338	121.1 (100), 93.1 (53.09), 107.1 (41.12);<br>121.1 (100), 93.1 (65.01), 136.2 (58.35);<br>121.2 (100), 93.1 (67.04), 136.2 (58.88)	NA	1336<br>(0.03±<br>0.01)	1338<br>(3.22±<br>0.01)	1337<br>(0.11±<br>0.01)
39	Citronellol<br>acetate	150-84-5	$C_{12}H_{22}O_2$	*MAc*	954 (954);<br>950 (950);<br>872 (881);<br>908 (908)	1355	81.1 (100), 95.1 (97.44), 69.1 (90.52);<br>95.1 (100), 81.1 (98.43), 69.1 (83.91);<br>81.1 (100), 95.1 (92.44), 69.1 (87.26);<br>81.1 (100), 95.1 (96.81), 69.1 (94.12)	1355<br>(0.14±<br>0.03)	1357<br>(2.81±<br>0.06)	1355<br>(0.02±<br>0.01)	1355<br>(0.19±<br>0.01)
40	Nerol<br>acetate	141-12-8	$C_{12}H_{20}O_2$	*MAc*	934 (935);<br>906 (908);<br>907 (907);<br>932 (932)	1367	69.2 (100), 93.1 (55.87), 68.2 (38.06);<br>69.2 (100), 93.2 (53.09), 68.1 (34.91);<br>69.2 (100), 93.2 (59.57), 68.1 (38.53);<br>69.1 (100), 93.1 (61.17), 68.1 (41.17)	1369<br>(3.57±<br>0.10)	1366<br>(0.25±<br>0.03)	1366<br>(0.03±<br>0.01)	1367<br>(0.73±<br>0.01)
41	Geranyl<br>acetate	16409-<br>44-2	$C_{12}H_{20}O_2$	*MAc*	951 (958);<br>916 (926);<br>914 (914);	1386	69.2 (100), 68.2 (36.97), 93.1 (34.17);<br>69.1 (100), 68.2 (38.07), 93.1 (35.94);<br>69.2 (100), 68.2 (36.12), 93.1 (35.08)	1376<br>(2.92±<br>0.04)	1386<br>(0.96±<br>0.02)	NA	1384<br>(0.39±<br>0.03)
42	Elemene, β-	515-13-9	$C_{15}H_{24}$	*SH*	918 (918);<br>919 (921);<br>929 (931)	1393	93.1 (100), 81.1 (86.08), 67.1 (83.02);<br>93.1 (100), 81.1 (82.01), 107.1 (72.88);<br>93.1 (100), 81.1 (82.01), 107.1 (74.12)	NA	1391<br>(0.05±<br>0.01)	1392<br>(1.32±<br>0.01)	1392<br>(2.78±<br>0.08)
43	Dodecanal	112-54-9	$C_{12}H_{24}O$	*AH*	884 (884);<br>885 (887);<br>922 (922)	1409	57.1 (100), 82.1 (78.51), 55.1 (72.68);<br>57.1 (100), 82.1 (89.26), 55.1 (70.25);<br>57.1 (100), 82.1 (98.40), 55.1 (86.26)	1410<br>(0.02±<br>0.01)	NA	1409<br>(0.02±<br>0.01)	1409<br>(0.10±<br>0.01)

44	Caryophyllene	87-44-5	$C_{15}H_{24}$	*SH*	923 (923); 952 (952); 937 (937); 950 (950)	1418	91.1 (100), 133.1 (94.72), 93.1 (83.53); 133.1 (100), 91.1 (92.12), 93.1 (87.47); 133.1 (100), 91.1 (95.02), 93.1 (82.61); 133.1 (100), 91.1 (91.34), 93.1 (85.56)	1418 (1.48± 0.02)	1418 (0.45± 0.01)	1419 (3.29± 0.01)	1418 (1.64± 0.03)
45	Copaene, β-	18252-44-3	$C_{15}H_{24}$	*SH*	945 (945)	1428	161.2 (100), 105.1 (34.42), 91.1 (30.29)	NA	NA	1428 (0.21± 0.01)	NA
46	Elemene, γ-	29873-99-2	$C_{15}H_{24}$	*SH*	922 (925)	1433	121.1 (100), 93.1 (60.39), 107.1 (43.11)	NA	NA	1433 (0.55± 0.01)	NA
47	Bergamoten, α-	17699-05-7	$C_{15}H_{24}$	*SH*	925 (944); 924 (947)	1430	119.1 (100), 93.1 (99.09), 91.1 (50.45); 119.1 (100), 93.1 (97.50), 91.1 (49.72)	1435 (0.11± 0.01)	NA	NA	1435 (0.16± 0.01)
48	Humulene	6753-98-6	$C_{15}H_{24}$	*SH*	914 (927); 921 (924); 914 (927); 942 (948)	1452	93.1 (100), 121.1 (36.02), 80.1 (29.55); 93.1 (100), 121.1 (36.77), 80.1 (27.13); 93.1 (100), 121.1 (35.41), 80.1 (29.10); 93.1 (100), 121.1 (35.99), 80.1 (29.46)	1452 (0.14± 0.04)	1452 (0.08± 0.01)	1452 (0.64± 0.05)	1452 (0.44± 0.01)
49	Geranyl propionate	105-90-8	$C_{13}H_{22}O_2$	*MAc*	887 (892)	1478	69.1 (100), 93.1 (75.96), 57.1 (52.10)	1476 (0.13± 0.02)	NA	NA	NA
50	Germacrene D	23986-74-5	$C_{15}H_{24}$	*SH*	888 (902); 946 (959); 922 (936)	1480	161.2 (100), 105.1 (50.92), 91.1 (47.97); 161.2 (100), 105.1 (49.83), 91.1 (47.17); 161.2 (100), 105.1 (49.62), 91.1 (47.63)	NA	1479 (0.04± 0.01)	1486 (13.04± 0.25)	1479 (0.36± 0.01)
51	Bicyclogermacrene	24703-35-3	$C_{15}H_{24}$	*SH*	920 (921); 920 (920); 923 (924); 918 (918)	1495	121.2 (100), 93.1 (65.23), 107.1 (48.60); 121.2 (100),93.1 (67.06), 107.1 (51.53); 121.1 (100), 93.1 (67.78), 107.1 (51.70); 121.1 (100), 93.1 (86.30), 107.1 (52.65)	1495 (0.13± 0.01)	1495 (0.33± 0.17)	1497 (2.03± 0.38)	1495 (0.14± 0.01)
52	Selinene, β-	17066-67-0	$C_{15}H_{24}$	*SH*	948 (958); 931 (935)	1489	105.1 (100), 93.2 (93.51), 107.1 (89.08); 93.2 (100), 105.1 (97.15), 107.1 (84.76)	NA	NA	1489 (2.42± 0.17)	1484 (0.10± 0.01)
53	Muurolene, α-	31983-22-9	$C_{15}H_{24}$	*SH*	878 (882)	1499	105.1 (100), 161.2 (77.77), 91.1 (52.76)	NA	1499 (0.02±	NA	NA

									0.01)		
54	Farnesene, α-	502-61-4	$C_{15}H_{24}$	*SH*	891 (898); 919 (922)	1508	93.1 (100), 107.2 (53.15), 91.1 (49.68); 93.1 (100), 107.1 (48.44), 91.1 (47.56)	NA	1509 (0.07± 0.01)	NA	1508 (0.66± 0.02)
55	Bisabolene, β-	495-61-4	$C_{15}H_{24}$	*SH*	903 (907)	1509	93.1 (100), 69.2 (76.32), 91.1 (44.11)	1509 (0.24± 0.07)	NA	NA	NA
56	Cubebol	23445-02-5	$C_{15}H_{26}O$	*OS*	923 (927)	1514	161.1 (100), 207.2 (83.35), 105.1 (44.34)	NA	1514 (0.16± 0.006)	NA	NA
57	Cadinene, δ-	483-76-1	$C_{15}H_{24}$	*SH*	862 (867); 912 (919); 912 (924); 902 (907)	1523	161.1 (100), 119.1 (81.47), 204.2 (67.84); 161.1 (100), 119.1 (63.70), 204.2 (53.74); 161.1 (100), 119.1 (63.87), 204.2 (57.56); 161.2 (100), 119.1 (65.44), 204.2 (60.50)	1523 (0.03± 0.01)	1523 (0.17± 0.01)	1523 (0.42± 0.01)	1523 (0.05± 0.01)
58	trans-Sesquisabinene hydrate	145512-84	$C_{15}H_{26}O$	*OS*	810(810)	1590	93.1 (100), 121 (71.77), 119 (61.72)	NA	NA	NA	1543 (0.02± 0.01)
59	Elemol	639-99-6	$C_{15}H_{26}O$	*OS*	943 (950); 950 (958); 933 (940)	1549	93.1 (100), 161.2 (97.07), 59.1 (93.10); 93.1 (100), 161.2 (94.41), 59.1 (82.73); 93.1 (100), 161.2 (89.99), 59.1 (88.31)	NA	1548 (0.42± 0.05)	1557 (16.67± 0.10)	1548 (0.19± 0.01)
60	Germacrene B	15423-57-1	$C_{15}H_{24}$	*SH*	908 (908)	1554	121.1 (100), 93.1 (90.38), 105.1 (79.82)	NA	NA	NA	1554 (0.03± 0.01)
61	Nerolidol	40716-66-3	$C_{15}H_{26}O$	*OS*	863 (863); 931 (938); 941 (950); 906 (911)	1564	69.2 (100), 93.1 (84.83), 107.1 (51.52); 69.2 (100), 93.1 (97.66), 107.1 (66.85); 69.2 (100), 93.1 (93.98), 107.1 (64.33); 69.2 (100), 93.1 (96.15), 107.2 (66.52)	1564 (0.02± 0.01)	1564 (0.80± 0.10)	1567 (1.53± 0.01)	1564 (0.09± 0.01)
62	Germacren D-4-ol	198991-79-6	$C_{15}H_{26}O$	*OS*	888 (893)	1574	81.1 (100), 161.1 (47.24), 105.1 (30.18)	NA	1574 (0.16± 0.01)	NA	NA
63	4,8,12-Trimethyl-	62235-06-7	$C_{16}H_{26}$	*AH*	878 (893); 915 (927)	1579	69.1 (100), 81.1 (57.51), 79.1 (27.69); 69.1 (100), 81.1 (50.60), 79.1 (18.43)	NA	1579 (0.12±	NA	1579 (0.14±

	1,3,7,11-tridecatetraene								0.01)		0.01)
64	Caryophyllene oxide	1139-30-6	$C_{15}H_{24}O$	*SO*	902 (904)	1580	93.1 (100), 79.1 (96.84), 91.1 (78.89)	1580 (0.11± 0.03)	NA	NA	NA
65	Cubenol	21284-22-0	$C_{15}H_{26}O$	*OS*	846 (882)	1646	161.1 (100), 93.1 (79.30), 105.1 (69.02)	NA	NA	1592 (0.20± 0.02)	NA
66	Cadinol, α-	5937-11-1	$C_{15}H_{26}O$	*OS*	895(897)	1635	161.2 (100), 204.2 (38.85), 105.1 (28.24)	NA	1640 (0.08± 0.01)	NA	NA
67	Eudesmol, epi-γ-	117066-77-0	$C_{15}H_{26}O$	*OS*	933 (951)	1620	189.2 (100), 162.1 (72.76), 204.2 (61.03)	NA	NA	1520 (1.70± 0.04)	NA
68	Eudesmol, γ-	1209-71-8	$C_{15}H_{26}O$	*OS*	932 (933)	1635	189.2 (100), 161.2 (95.46), 204.2 (78.66)	NA	NA	1635 (5.69± 0.20)	NA
69	Agaruspirol	1460-73-7	$C_{15}H_{26}O$	*OS*	898 (950)	1646	161.2 (100), 119.2 (48.14), 107.1 (40.38)	NA	NA	1640 (0.31± 0.04)	NA
70	Eudesmol, β-	473-15-4	$C_{15}H_{26}O$	*OS*	952 (960)	1656	149.2 (100), 59.2 (78.76), 164.2 (44.78)	NA	NA	1656 (8.58± 0.05)	NA
71	Eudesmol, α-	473-16-5	$C_{15}H_{26}O$	*OS*	940 (951)	1659	149.2 (100), 161.2 (98.27), 204.2 (87.13)	NA	NA	1659 (3.62± 0.04)	NA
72	Bisabolol	515-69-5	$C_{15}H_{26}O$	*OS*	909 (999); 929 (930)	1685	109.1 (100), 119.1 (85.04), 69.1 (70.55); 109.1 (100), 119.1 (82.45), 69.1 (78.55)	1685 (0.07± 0.01)	NA	NA	1685 (0.11± 0.01)
73	Elemyl acetate	60031-93-8	$C_{17}H_{28}O_2$	*MAc*	889 (917)	1675	161.1 (100), 93.1 (93.07), 107.2 (76.68)	NA	NA	1678 (0.18±	NA

										0.01)	
74	Germacra-4(15),5,10(14)-trien-1β-ol	81968-62-9	$C_{15}H_{24}O$	*OS*	941 (944)	1694	91.1 (100), 109.1 (88.64), 159.1 (87.25)	NA	NA	1686 (0.32± 0.03)	NA
75	Farnesol	106-28-5	$C_{15}H_{26}O$	*OS*	857 (857)	1722	69.2 (100), 81.1 (39.53), 93.0 (30.20)	NA	1722 (0.03± 0.01)	NA	NA
76	Farnesal	502-67-0	$C_{15}H_{24}O$	*OS*	886 (889)	1730	69.2 (100), 84.2 (51.27), 81.1 (27.62)	NA	NA	1743 (0.05± 0.01)	NA
77	Neophytadiene	504-96-1	$C_{20}H_{38}$	*DH*	879 (879)	1840	95.0 (100), 68.1 (79.29), 67.1 (68.76)	NA	NA	NA	1840 (0.02± 0.01)
78	Farnesol acetate	264.208931	$C_{17}H_{28}O_2$	*MAc*	881 (881)	1834	69.2 (100), 93.1 (56.81), 81.1 (35.71)	NA	NA	1843 (0.03± 0.01)	NA
79	Citroptene	487-06-9	$C_{11}H_{10}O_4$	*AK*	873 (873)	1916	206 (100), 178.2 (99.43), 163 (57.22)	NA	NA	NA	1972 (0.02± 0.001)
80	Phytol	150-86-7	$C_{20}H_{40}O$	*OD*	929 (931); 911 (913); 922 (923); 929 (931)	2113	71.1 (100), 123.2 (43.38), 81.1 (33.37); 71.1 (100), 123.1 (44.94), 57.1 (38.08); 71.1 (100), 123.1 (44.15), 81.2 (38.00); 71.1 (100), 123.2 (47.34), 81.2 (39.51)	2113 (1.62± 0.12)	2113 (0.21± 0.02)	2112 (0.39± 0.01)	2113 (0.78± 0.04)
		Monoterpene hydrocarbons						48.63	5.20	14.48	76.15
		Oxygenated monoterpenes						42.74	89.52	3.91	13.52
		Sesquiterpene hydrocarbons						2.13	1.36	27.14	6.60
		Oxygenated sesquiterpenes						0.21	1.66	38.87	0.42
		oxygenated diterpenes						1.61	0.21	0.39	0.79
		Others						0.28	0.02	0.10	0.52
		Total						95.78	97.97	84.88	97.99

[a]Class of chemical compounds: *MH* monoterpenic hydrocarbon, *MO* monoterpenic oxide, *MA* monoterpenic aldehyde, *OM* monoterpenic alcohol, *MAc* monoterpenic acetate, *SH* sesquiterpenic hydrocarbon, *SA* sesquiterpenic aldehyde, *OS* sesquiterpenic alcohol, *DH* diterpens, *OD* oxygenated diterpenes, *AH* acyclic hydrocarbon, *AK* acyclic ketone, *AE* acyclic ester, *OA* acyclic alcohol

[b]Matching scores of compounds reported ≥80% based on the mass spectra in NIST library database and in the order of *C. limon* EO (CL), *C. hystrix* EO (CH), *C. microcarpa* EO (CM), *C. pyriformis* EO (CP).

[c]Fragmentation patterns reported in order of *C. limon, C. hystrix, C. microcarpa, and, C. pyriformis*.

[d]Retention index (RI) values calculated using Van den Dool and Kratz equation with reference to RI values from NIST WebBook, and Adams (2007) within the range of ±10.

[e]Relative percentage abundance calculated on the basis of TIC (Total Ion Chromatogram) area as the percentage of total TIC area.

NA: compound not available.

Table S2. Yields of four *Citrus* spp. leaf essential oils.

***Citrus* spp.**	**Yields (% w/w)**
C. hystrix	0.72
C. limon	0.41
C. pyriformis	0.28
C. microcarpa	0.49

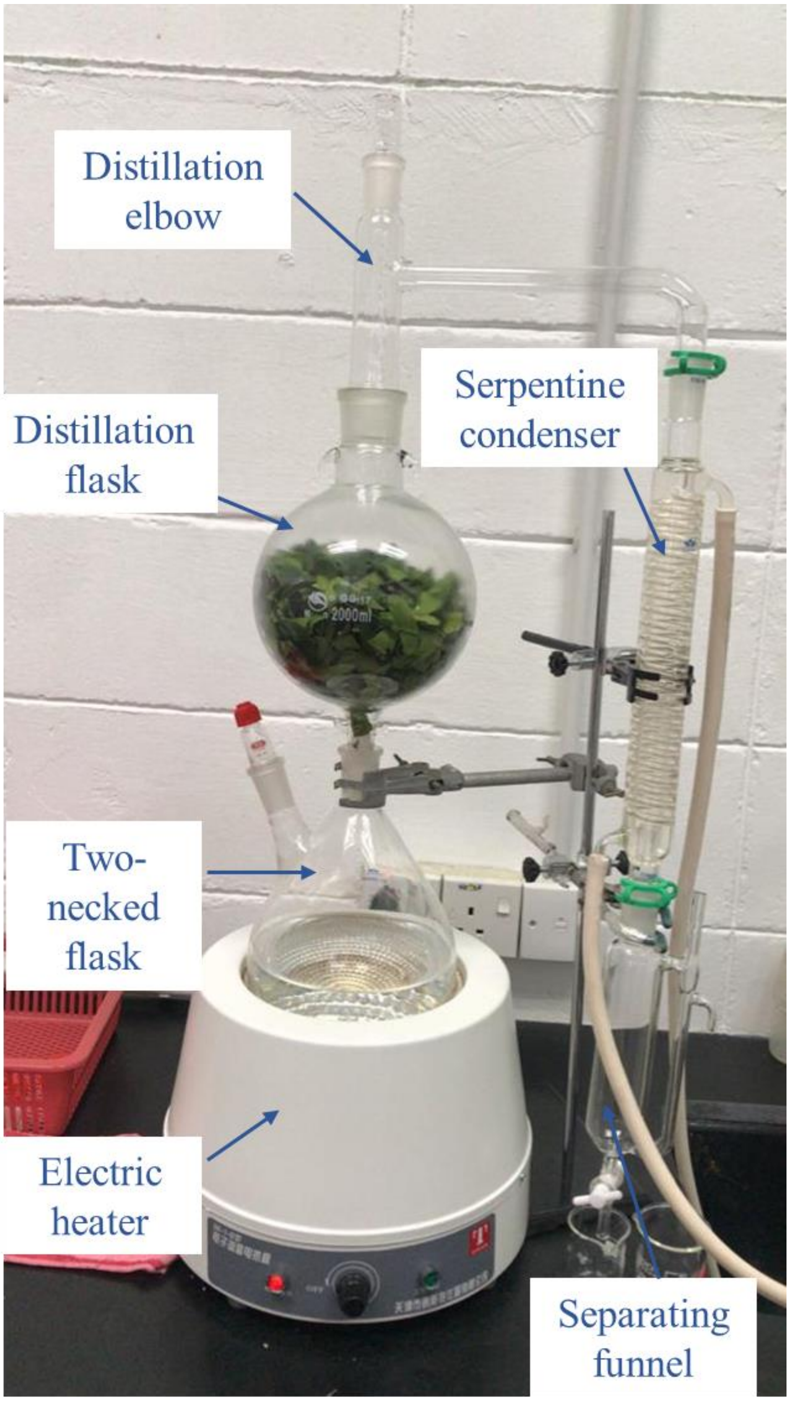

Figure S1. Steam distillation equipment used for the extraction of *Citrus* spp. leaf essential oils.
